# Influence of Bloat Control on Relocation Rules Automatically Designed via Genetic Programming

**DOI:** 10.3390/biomimetics11010083

**Published:** 2026-01-21

**Authors:** Tena Škalec, Marko Đurasević

**Affiliations:** Faculty of Electrical Engineering and Computing, University of Zagreb, Unska 3, 10000 Zagreb, Croatia; tena.skalec@fer.hr

**Keywords:** container relocation problem, genetic programming, hyper-heuristics, bloat, relocation rules, simplification, parsimony pressure

## Abstract

The container relocation problem (CRP) is a critical optimisation problem in maritime port operations, in which efficient container handling is essential for maximising terminal throughput. Relocation rules (RRs) are a widely adopted solution approach for the CRP, particularly in online and dynamic environments, as they enable fast, rule-based decision-making. However, the manual design of effective relocation rules is both time-consuming and highly dependent on problem-specific characteristics. To overcome this limitation, genetic programming (GP), a bio-inspired optimisation technique grounded in the principles of natural evolution, has been employed to automatically generate RRs. By emulating evolutionary processes such as selection, recombination, and mutation, GP can explore large heuristic search spaces and often produces rules that outperform manually designed alternatives. Despite these advantages and their inherently white-box nature, GP-generated relocation rules frequently exhibit excessive complexity, which hinders their interpretability and limits insight into the underlying decision logic. Motivated by the biomimetic observation that evolutionary systems tend to favour compact and efficient structures, this study investigates two mechanisms for controlling rule complexity, parsimony pressure, and solution pruning, and it analyses their effects on both the quality and size of relocation rules evolved by GP. The results demonstrate that substantial reductions in rule size can be achieved with only minor degradation in performance, measured as the number of relocated containers, highlighting a favourable trade-off between heuristic simplicity and solution quality. This enables the derivation of simpler and more interpretable heuristics while maintaining competitive performance, which is particularly valuable in operational settings where human planners must understand, trust, and potentially adjust automated decision rules.

## 1. Introduction

Improving the quality of shipping services is a continual objective in global trade [1]. One major operational bottleneck arises in cargo bays, where shipping containers are temporarily stored before being loaded onto vehicles for onward transport. Owing to limited space, containers are stacked vertically, and cranes can retrieve only those containers located at the top of a stack. Consequently, accessing any container beneath the topmost position requires all containers above it to be relocated to other stacks. As containers cannot be presorted according to their departure order due to incomplete or unavailable information [2], multiple relocations during the retrieval process are often unavoidable. The operational objective in such environments is, therefore, to minimise the number of container relocations, as these constitute unproductive and wasteful movements. This optimisation problem is commonly referred to as the container relocation problem (CRP), also known as the block relocation problem [3].

The CRP has been shown to be an NP-hard problem [4], implying that no algorithm can efficiently compute optimal solutions for all instances. Consequently, a wide range of solution approaches has been proposed to address the CRP [5]. One prominent class of approaches consists of exact methods, including dynamic programming [6,7], branch-and-bound techniques [8,9], and integer programming formulations [10,11]. While these methods can guarantee optimality, they are typically computationally expensive and often require substantial time to solve even a single problem instance. As an alternative, numerous metaheuristic approaches have been developed, such as genetic algorithms [1], simulated annealing [12], and greedy randomised adaptive search procedures (GRASP) [13,14]. Although these methods generally offer improved scalability compared to exact approaches, they may still incur considerable computational costs. Moreover, metaheuristics are often unsuitable for settings in which only partial or incomplete information is available, such as online or dynamic environments. For these reasons, simple heuristic methods known as relocation rules (RRs) are frequently employed to efficiently solve CRPs under practical operational constraints.

RRs are simple greedy procedures that construct solutions to the CRP in an iterative manner. Rather than generating a complete solution in advance, they begin with an empty solution and determine which action to perform at each decision point. In the context of the CRP, a decision point arises whenever a container must be relocated from its current stack to an alternative stack. At each such decision point, the RR evaluates all feasible destination stacks according to a greedy criterion, typically based on features such as stack height, the number of containers that would become blocked, or other related measures. The container is then relocated to the stack deemed optimal under this evaluation strategy. Because RRs neither search the entire solution space nor construct the full solution upfront, they can generate solutions very efficiently and readily adapt to changes in the problem environment, making them particularly well suited for online and dynamic settings [15,16].

Designing effective heuristics is, however, a challenging and labour-intensive task that typically requires substantial domain expertise. To alleviate this burden, hyper-heuristic approaches have been proposed [17] to automatically generate suitable heuristics for a wide range of combinatorial optimisation problems, including scheduling [18,19], routing [20,21,22], bin packing [23], and the CRP [24,25]. Among the various hyper-heuristic techniques, genetic programming (GP) has emerged in the literature as a particularly well-suited and effective method for this purpose [26,27].

From a biomimetic perspective, GP belongs to a broader class of optimisation methods inspired by natural evolutionary processes [28,29]. By emulating mechanisms such as variation, competition, and selection, GP mirrors the way biological systems adapt their structures and behaviours to the environment over successive generations [30]. Unlike many other metaheuristic methods that optimise a fixed set of variables representing a concrete solution, GP evolves tree-like structures that encode complex formulae and can be applied across different contexts [29]. When used as a hyper-heuristic, these evolved structures represent heuristics tailored to solving a specific combinatorial optimisation problem. Viewed in this way, the heuristics generated via GP can be regarded as artificial artefacts shaped through an evolutionary design process, rather than explicitly engineered by human experts, which closely aligns GP with core biomimetic principles [26,31]. This biomimetic foundation is particularly relevant in the context of heuristic generation. Biological systems are often characterised by simple, robust, and reusable decision mechanisms that emerge through evolution, rather than through centralised design [32]. Similarly, GP facilitates the emergence of decision rules through evolutionary pressure, allowing effective heuristics to arise from combinations of relatively simple components [28].

Despite being widely praised for its ability to produce white-box solutions, GP often generates models that are highly complex and difficult to interpret. This issue, commonly referred to as bloat, arises when GP produces overly large and intricate solutions in situations where much simpler alternatives could achieve comparable performance while remaining easier to understand [28]. During the evolutionary process, solution structures tend to grow in size, frequently resulting in unnecessary complexity. Bloat gives rise to several well-documented problems in GP, including an increased risk of overfitting [33] and a significant loss of interpretability [34]. The latter is particularly problematic in the context of hyper-heuristic methods, for which the evolved heuristics are intended to guide decision-making processes but may themselves become opaque and difficult to analyse [35]. The ability to understand and explain how such heuristics operate and construct solutions is essential for building human trust and enabling their adoption in real-world applications. Consequently, advancing research on methods for obtaining more interpretable solutions is imperative, not only within GP but also across artificial intelligence more broadly [36,37].

Given that the topics of solution simplification and interpretability have been rarely studied in the context of hyper-heuristics, this study investigates methods for reducing the tree size of GP-generated solutions when evolving relocation rules (RRs) for the CRP. Two complementary approaches were explored. The first, parsimony pressure [38], aims to limit solution growth during the evolutionary process itself. The second, pruning [39], simplifies the GP solution after the evolutionary run has completed. Both methods were evaluated in terms of their ability to reduce the solution size while minimally affecting solution quality. Furthermore, an additional analysis on the structure of solutions was performed to analyse what effect the two methods have on the complexity and interpretability of the generated solutions.

The remainder of the paper is organised as follows. Section 2 provides an overview of related literature. Section 3 presents the CRP and its main variants. Section 4 describes the use of GP for the automatic design of RRs for the CRP and introduces the methods explored in this study for controlling tree size, namely parsimony pressure and pruning. The experimental study applying these methods is detailed in Section 5, including an overview and comparison of results. Section 6 analyses the effect of tree size control methods on the structure of the evolved solutions. Finally, Section 7 concludes the paper and discusses directions for future research.

## 2. Literature Review

Since its first formal definition in [3], the CRP has attracted significant attention from the scientific community due to its practical relevance, leading to the development of numerous solution methods and problem variants [5]. Approaches to solving the CRP can be broadly categorised as exact or heuristic. Among the most widely used exact methods are the A* algorithm [40,41,42], branch-and-bound techniques [11,43], and dynamic programming [44]. Although these exact approaches can solve all benchmark instances optimally [10], their high computational complexity often renders them impractical for real world applications.

Heuristic methods are often employed as an alternative when solution optimality is less critical but rapid computation is required. These methods can generally be divided into metaheuristics and problem-specific heuristics. Metaheuristics are general-purpose optimisation algorithms that can be readily adapted to a wide range of optimisation problems, and many have been applied to various CRP variants [5]. Among these, GRASP is the most commonly used metaheuristic in the literature for CRP [13,45], although other approaches, such as simulated annealing [12], tabu search [46], and genetic algorithms [1], have also been applied. While metaheuristics do not guarantee optimal solutions for all instances, most studies show that they can produce high-quality solutions in a fraction of the time required for exact methods.

In contrast to metaheuristics, problem-specific heuristics are designed to address a particular problem, such as the CRP. These heuristics, commonly referred to as RRs, typically solve the problem in a constructive manner. Rather than exploring the entire solution space, RRs make decisions sequentially at each decision point, for example, determining to which stack a blocking container should be relocated. Various strategies can be employed within RRs, such as relocating the container to the lowest stack [47], moving it to the stack that would introduce the fewest additional blockages [47], or other context-specific strategies [40,48]. Although RRs generally do not achieve the solution quality of exact methods or metaheuristics, their low computational complexity and ability to react immediately to changing conditions make them well suited to environments with incomplete information or strict time constraints [44,49].

Regarding problem variants, the standard restricted CRP has been most commonly studied in the literature, particularly in the early years [8,50]. More recently, research attention has shifted towards the unrestricted variant [11,51,52], which, although more complex to solve, has been shown to yield better performance compared to the restricted version. Beyond these standard variants, numerous studies have proposed additional CRP variants to better capture real-world scenarios. These include the multibay CRP, in which relocations can occur between multiple bays [47]; the dynamic CRP, where additional containers may arrive in the yard over time [46,53]; the online CRP, in which complete retrieval information is not available [15,16]; and the real-time CRP, with which relocation decisions must be made rapidly [54].

The automated design of RRs for the CRP was first explored in [24], where the authors aimed to minimise the total energy consumption of all crane operations. The proposed RR, called GRH, employed a fixed strategy to select the destination stack for each container but included several free parameters that could be optimised. A genetic algorithm was then used to determine the optimal values for these parameters. While this approach produced promising results, its main limitation was the need to predefine a fixed RR structure, which requires expert knowledge and constrains the potential solutions that can be discovered. As an alternative, GP was employed to automatically design RRs in [25]. Unlike the approach of [24], GP does not impose restrictions on the structure of the destination selection strategy, allowing the algorithm to freely evolve rules tailored to the problem at hand. The results demonstrated that GP-designed RRs can achieve significantly better performance than manually designed rules. Consequently, this method has been extended to other problem variants, including the multibay CRP and CRP with duplicate container IDs [55], energy minimisation [56], and the online CRP [57]. Beyond exploring different problem variants, GP has also been combined with complementary techniques to further enhance the performance of evolved RRs, such as the rollout algorithm [58], ensembles of RRs [59], multitask learning [60], and the design of RRs with improved look-ahead capabilities [61].

The phenomenon of expression growth without a corresponding increase in fitness in GP was first observed in the early 1990s and documented in [62], eventually being termed bloat. In [63], the authors argue that the exponential shape of the underlying search space in GP, combined with selection pressure, drives the algorithm toward larger solutions, concluding that bloat is therefore intrinsic to GP. Various methods for controlling bloat have been explored, with parsimony pressure being among the most widely used. In [38], the authors discuss the challenge of selecting the parsimony coefficient and introduce a generalised approach to dynamically determining the optimal coefficient. Another approach involves simplifying the expression trees generated via GP. This can be achieved through algebraic simplification during evolution [64], by replacing expressions with simpler equivalents that maintain numerical equivalence over a suitable range of inputs [65], or via subtree pruning, where a subtree is replaced with its expected value (calculated over a suitable input range) as long as this does not significantly alter the overall output of the tree [66]. A variation of this approach, called prune and plant, was proposed in [67], in which the pruned portion of the tree is reintroduced as a new individual in the population to evolve into a new solution. In addition, several genetic operators have been developed specifically to mitigate bloat, including hoist mutation [68], shrink mutation [69], and homologous crossover [70]. These techniques collectively aim to control the growth of GP solutions without compromising their effectiveness.

Although bloat is a well-known issue in GP, it has rarely been investigated in the context of hyper-heuristic methods. In [39], the authors applied two simplification techniques, pruning and algebraic simplification, to dispatching rules generated for the unrelated machines environment. Their experimental analysis showed that pruning was the more effective method, significantly reducing the size of the expressions, whereas algebraic simplification only produced minor reductions. In [71], the authors extended algebraic simplification with additional operators that incorporate problem-specific information. The results demonstrated that this approach can substantially reduce the expression size while having minimal impact on performance. Further extending this work, in [72] the authors proposed new numerical and behavioural simplification operators for the dynamic job shop-scheduling problem, providing additional tools for controlling solution complexity in hyper-heuristic environments.

## 3. The Container Relocation Problem

In the CRP, a set of *N* containers is stored in a container yard with a limited capacity, requiring them to be arranged in stacks, with one container placed on top of another [6]. The containers must then be retrieved via a gantry crane according to a predefined order, specified with unique container IDs, where smaller IDs indicate that a container should be retrieved earlier. The container with the smallest ID in the yard at any given moment is referred to as the target container, as it is the next container to be retrieved. If the target container is located at the top of its stack, it can be retrieved immediately via the crane. However, if other containers are stacked above the target container, these blocking containers must first be relocated to other stacks. The stack from which a container is moved is typically called the origin stack, while the stack to which it is relocated is the destination stack. Each stack has a maximum allowable height of *H*, meaning that no more than *H* containers can be placed on a stack at any time. Consequently, when relocating a container, the destination stack must be chosen from those with available capacity.

Two main CRP variants are distinguished by relocation constraints. In the restricted CRP, only containers stacked above the target container can be relocated, whereas in the unrestricted CRP, containers from any stack may be moved. Feasible solutions to the CRP consist of sequences of relocations and retrievals that ensure that all containers are retrieved in the predetermined order. The preferred solutions are those that optimise a specific objective, such as minimising the total crane operation time [47], the total energy consumed via crane operations [24], or, most commonly, the total number of relocations.

**Figure 1:**
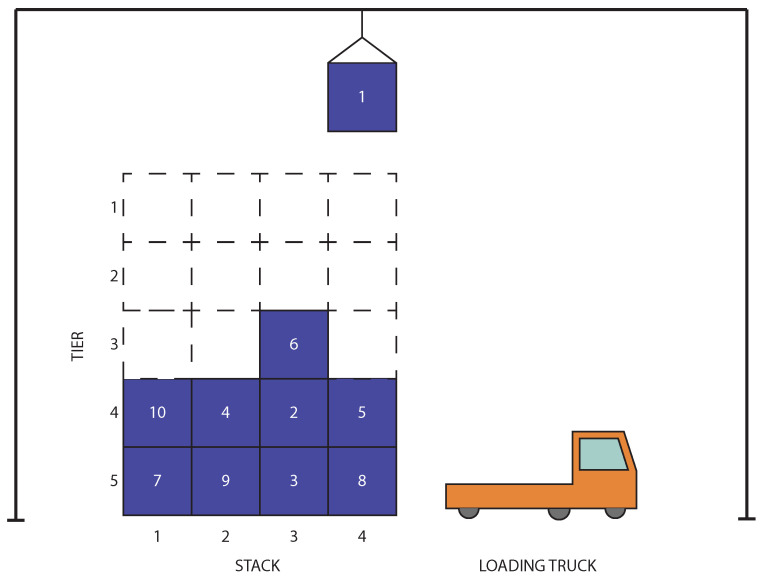
Illustration of the container relocation problem (CRP): a gantry crane retrieves containers for loading onto outbound transport in ascending order of priority denoted with container IDs. Container 1 is currently being loaded onto a moving truck, and container 2 is the next in the retrieval sequence, blocked by container 6, which will require relocation in order to perform the retrieval of container 2.

Figure 1 illustrates an example CRP instance. In this scenario, the container with ID 1 is currently being retrieved via the gantry crane and will be placed on the loading truck. The next container to be retrieved is 2, which is blocked by container 6 and must therefore be relocated. While container 6 could, in principle, be moved to any other stack, relocating it to stacks 2 or 4 would cause it to block containers with smaller IDs. The optimal decision in this case is to relocate container 6 to stack 1, which contains only containers with larger IDs. This ensures that no container is blocked by container 6, after which container 2 becomes accessible and can be retrieved. The process continues in this manner until all containers have been retrieved from the yard.

## 4. Automated Design of Relocation Rules

### 4.1. Genetic Programming

GP is an evolutionary optimisation method that emulates mechanisms of natural selection and evolution [28,62]. The outline of the GP algorithm used in this study is given in Algorithm 1. Initially, a population of individuals, each representing a potential solution, is generated randomly. The fitness of each individual is then evaluated using a predefined fitness function. The population evolves through the creation of offspring that inherit attributes from parent individuals, occasionally subject to random mutations. In each iteration, a three-tournament selection is performed: three individuals are randomly chosen from the population, and the two with the highest fitness participate in the crossover. Crossover combines information from both parents to produce a new child individual, which may then undergo mutation with a certain probability. Mutation introduces random changes, helping the algorithm maintain diversity and avoid suboptimal local minima. The child individual is evaluated using the fitness function and replaces the worst individual from the tournament, the one that did not participate in the crossover. This process is repeated until a predefined number of fitness evaluations is reached, at which point the algorithm terminates, and the best individual from the final population is returned as the final solution.
**Algorithm 1:** Steady-state GP with tournament selection
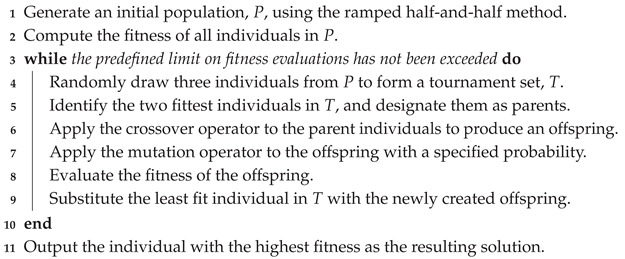


**Figure 2:**
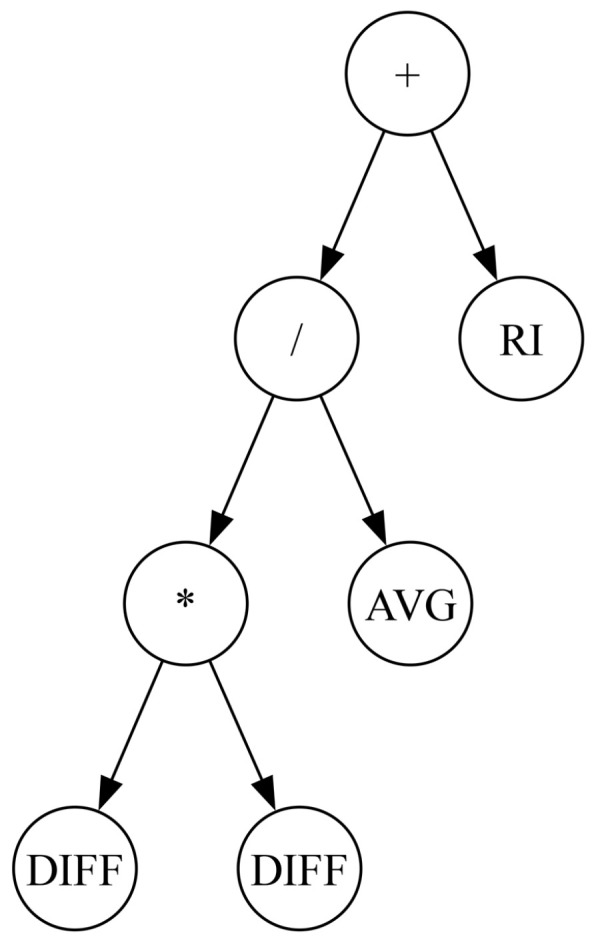
Tree representation of an expression in GP. This example has three function nodes (+, −, and ∗), and four terminal nodes (RI, AVG, and two DIFF nodes).

As described above, the evolutionary process in GP is conceptually similar to that of standard genetic algorithms (GAs). The key difference lies in the solution representation. In GP, individuals are represented as expression trees of variable sizes. The internal nodes of these trees, called function nodes, consist of mathematical or problem-specific operators, while the leaves, called terminal nodes, represent problem-specific variables or constants. Unlike GAs, for which all solutions have a fixed size and structure, GP allows solutions to vary in size and structure, constrained only by a maximum allowed tree depth. This restriction sets the upper limit on the number of nodes in the expression tree, but GP can evolve smaller solutions through crossover and mutation operations. Figure 2 illustrates an example expression tree representing a potential GP solution, which can be decoded into the following expression:

$$RI+\frac{AVG}{DIFF*DIFF}$$

Here, +, ∗, and / represent function nodes, whereas DIFF, AVG, and RI represent problem-specific variables (defined in the next section).

### 4.2. Designing Relocation Rules with Genetic Programming

Relocation rules (RRs) consist of two components: the relocation scheme (RS) and the priority function (PF) [25]. The RS is responsible for performing container retrievals and relocations in a manner that guarantees a feasible solution. It first checks whether the target container is on top of its stack and can therefore be retrieved immediately. If not, the RS relocates the blocking containers to other stacks. To determine the most suitable destination stack for a relocation, the RS identifies all feasible stacks with available capacity. The PF is then used to assign a priority value to each potential destination stack based on the current state of the system. The stack with the lowest priority value is selected as the destination stack for the relocation.

At this stage, the behaviour of the RS depends on whether restricted or unrestricted relocations are allowed. In the restricted variant, each blocking container is moved directly to the selected destination stack. In the unrestricted variant, the RS first checks whether placing the blocking container onto the destination stack would block the current top container. If this is the case, the RS attempts to prevent new blockages by relocating the top container of the destination stack to an alternative stack where it would not create further blockages, provided that such a relocation is feasible. This process is repeated as long as blockage-free relocations are possible. Once no further such relocations can be performed, the blocking container is finally placed on the destination stack. After all blocking containers have been relocated, the target container becomes accessible for retrieval. This procedure is repeated until all containers have been retrieved from the cargo bay. A formal outline of the RS is provided in Algorithm 2.
**Algorithm 2:** Relocation scheme
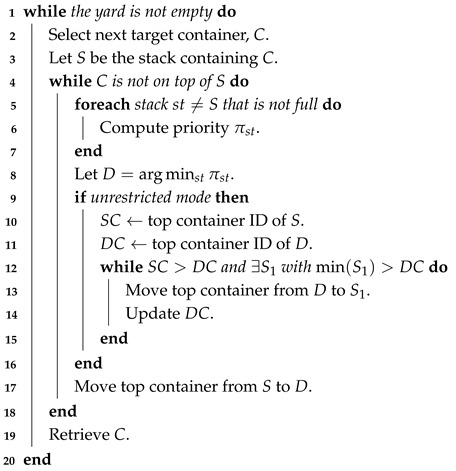


In this work, the RS follows a standard, manually defined structure, allowing GP to focus exclusively on evolving the PF. Designing an effective PF, however, is considerably more complex, as it must combine multiple system properties to assign meaningful priority values for all relocation decisions. Determining how to combine this problem information manually is challenging, making PFs an ideal target for automatic generation via GP. To enable GP to generate PFs, it is necessary to define the building blocks, namely the sets of function and terminal nodes that GP will use in constructing PFs. In this study, the function and terminal sets are summarised in Table 1. Only basic arithmetic operators are included in the function set, while the terminal set contains both simple variables (e.g., SH, EMP) and more complex problem-specific measures (e.g., RI, DIFF). Both the function and terminal sets were previously optimised to achieve the best possible results [25].

### 4.3. Controlling Tree Size

Expression trees evolved by GP can become large and difficult to interpret, which may limit their practical applicability due to reduced trust in the decision-making process [73]. Within these trees, certain subexpressions may contribute very little to the solution relative to their size. For instance, a complex or deep branch may have minimal impact on fitness or may even be entirely redundant. Several studies have observed that GP often reaches a stage where trees grow rapidly without corresponding improvements in fitness [38]. This excessive growth, known as bloat, is one of the most significant challenges associated with GP. Various simplification techniques can be applied to GP expression trees to control bloat, improving both solution interpretability and generalisation ability. Online simplification techniques are applied during the evolutionary process, whereas offline simplification techniques are applied to the final solutions after GP has completed its run.

Parsimony pressure is a simple and widely used online method for controlling bloat in GP [38]. This technique curbs tree overgrowth by introducing a penalty that is typically proportional to the complexity of the solution, measured by tree size, and controlled by a parameter called the parsimony coefficient (PC). Formally, parsimony pressure is defined as

$$fitness_{p}\left(sol\right)=fitness_{u}\left(sol\right)+PC*size\left(sol\right)$$

where $fitness_{p}\left(sol\right)$ is the penalised fitness of a solution sol, $fitness_{u}\left(sol\right)$ is the original fitness, and size(sol) denotes the number of nodes in the expression tree. This formulation biases selection toward smaller trees, although a larger tree can still be chosen if its original fitness is high enough to compensate for its size. The parsimony coefficient regulates the strength of this bias: a larger PC more strongly suppresses tree growth, while a smaller PC may be insufficient to control bloat. However, if PC is set too high, the algorithm may converge to overly small trees with poor fitness, whereas if it is too low, large and unnecessarily complex solutions may persist.

Determining an appropriate parsimony coefficient (PC) is typically problem-dependent and requires experimentation. To address this, this study employs an adaptive parsimony pressure approach, which adjusts the PC dynamically based on the current state of the population [38]. In this method, the following value is calculated for the current population:

$$\kappa =\left\{\begin{array}{cc} \frac{\mathrm{Cov}(f,c)}{\mathrm{Var}(c)}, & \mathrm{if}\;\mathrm{Var}(c)>0,\\ 0, & \mathrm{otherwise} \end{array}\right.,$$

where Cov(f,c) represents the covariance between the solution fitness and the solution size, defined as follows:

$$\mathrm{Cov}\left(f,c\right)=\frac{1}{N}\sum_{i=1}^{N}\left(f_{i}-\bar{f}\right)\left(c_{i}-\bar{c}\right),$$

while Var(c) denotes the variance of solution sizes:

$$\mathrm{Var}\left(c\right)=\frac{1}{N}\sum_{i=1}^{N}{\left(c_{i}-\bar{c}\right)}^{2},$$

with $\bar{c}$ and $\bar{f}$ denoting the average values of solution sizes and fitness values in the current population:

$$\bar{f}=\frac{1}{N}\sum_{i=1}^{N}f_{i}\;\bar{c}=\frac{1}{N}\sum_{i=1}^{N}c_{i}.$$

Finally, the new PC value is calculated by smoothing between the current PC value and κ, as follows:

PC=(1−s)PC+sκ,

where *s* represents the smoothing factor. The parsimony coefficient adapts dynamically based on the covariance between fitness and tree complexity, penalising larger trees only when additional complexity does not result in a proportional improvement in fitness.

Pruning [39] is an offline simplification method that removes the branches of the expression tree that contribute least to solution quality by replacing them with neutral elements. A neutral element is defined as one that, when combined with another element using a mathematical operator, leaves the other element unchanged. For example, 0 for addition or subtraction and 1 for multiplication or division. To identify potentially redundant branches, pruning iterates through each node of the expression tree in a breadth-first manner, starting from the root. At each node, the method attempts to replace it with the appropriate neutral element according to its parent operator. While this reduces tree size, it can alter the global semantics of the expression, so the modified tree is evaluated again using the fitness function. In this study, pruning decisions are governed by a relative fitness change (RFC) criterion, defined as follows:

(1) $$RFC=\frac{fitness_{p}-fitness_{o}}{fitness_{o}},$$

where $fitness_{p}$ is the fitness of the pruned tree, and $fitness_{o}$ is the fitness of the original GP-generated tree. A pruning step is accepted if the resulting RFC is below a user-defined threshold; otherwise, it is rejected. Absolute values are not used, as negative RFC values indicate that the pruned tree is better than the original, which is desirable. For example, a threshold of 0.05 allows a maximum deterioration of 5% in solution quality. The pruning procedure continues until all nodes in the tree have been evaluated. The outline of this procedure is provided in Algorithm 3.
**Algorithm 3:** Expression tree pruning using relative fitness change (RFC)
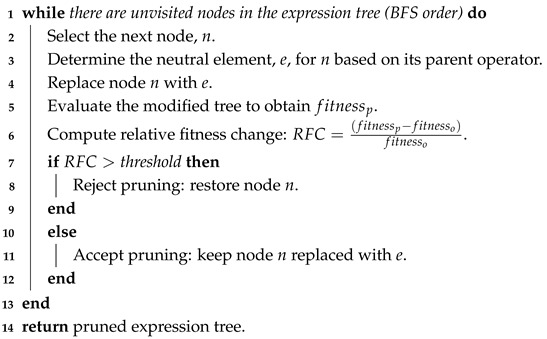


## 5. Experimental Study

### 5.1. Experimental Setup

To evaluate the effectiveness of parsimony pressure and pruning in reducing the complexity of RRs, we use the GP algorithm described in Section 4.1 to generate new rules. The GP parameter values are summarised in Table 2 and were extensively fine-tuned in a previous study [25]. Since multiple genetic operators are defined for both mutation and crossover, a random operator is selected for each operation in every iteration. Additionally, while a maximum tree depth of 5 typically yields the best results, experiments in this study are also conducted with a tree depth of 7 to assess the effectiveness of the simplification methods on both smaller and larger expression trees.

To objectively evaluate the performance of GP, two datasets are required: a training set and a test set. The training set is used during the evolutionary process, where GP searches for effective RRs for the given problem. However, since the goal is to generate RRs that generalise well to other problems, their performance must also be assessed on a separate test set, containing instances not seen during training. In this study, the original Caserta dataset [6] is used as the test set to evaluate generalisation. It contains 840 instances, with the number of stacks and containers per stack ranging from 3 to 10. All stacks are assumed to have the same initial height, *h*, with a maximum allowed height of h+2. For the training set, a new problem set of the same size and with similar properties was generated. An individual RR is evaluated by applying it to each instance in the dataset and counting the total number of container relocations performed. The overall fitness of the individual is then defined as the sum of relocations across all instances, with lower values indicating better performance.

To analyse the effectiveness and sensitivity of the simplification methods, experiments were conducted across a range of parameter values, selected based on preliminary experiments to provide a meaningful trade-off between solution quality and tree size. For standard parsimony pressure, PC values of 1, 10, 30, 50, 70, and 100 were considered. These values were chosen relative to the observed fitness scale and solution complexity. The best solutions typically have fitness values around 24,000–25,000 relocations, while tree sizes vary from approximately 10 to 200 nodes, depending on the maximum depth. Smaller PC values, therefore, impose only a minor penalty (well below 1% of fitness), whereas larger PC values can impose penalties comparable to the fitness itself for very large trees, strongly favouring more compact solutions. For adaptive parsimony pressure, experiments were conducted using different initial PC values and smoothing parameters, *s*. In the results, we report the most representative outcomes for an initial PC value of 1 and smoothing parameters of 0.001, 0.01, and 0.05.

For the pruning method, thresholds of 0.01, 0.03, 0.05, 0.1, and 0.2 were considered, corresponding to allowing a relative fitness deterioration of approximately 1% to 20% compared to the original solution. Larger thresholds were not evaluated, as preliminary experiments indicated that values above 0.2 caused substantial degradation in solution quality. In general, smaller values of the parsimony coefficient or pruning threshold prioritise solution quality, whereas larger values increasingly favour simpler, more compact solutions.

Experiments were conducted for both the restricted and unrestricted CRP variants to assess whether the effectiveness of the simplification methods depends on the problem variant, as different relocation strategies may be used in the two cases. To obtain reliable data for statistical analysis, each experiment was repeated 30 times, with the best individual from each run recorded. These 30 individuals were then evaluated on the test set to assess their generalisation performance. From these results, the minimum, maximum, and median fitness values were calculated and are reported in Section 5. Additionally, the Mann–Whitney test was applied at a significance level of 0.05 to determine whether differences between individual results are statistically significant.

The experiments were performed on a PC running Windows 11 Education, using the CPU AMD Ryzen Threadripper 7980X (3.20 GHz) with 64 cores, and with 256 GB of RAM. The software code was written in C++, based on the Evolutionary Computing Framework (ECF), version 1.4.2 [74].

### 5.2. Experimental Results

In this section, we first analyse the results of each simplification method individually, and then we provide a comparative evaluation of the two methods.

#### 5.2.1. Results for Parsimony Pressure

Table 3 presents the results for the restricted CRP variant when the parsimony pressure (PP) simplification method is applied. The table compares GP performance without and with PP in terms of fitness (i.e., the total number of container relocations) and tree size. For each tree depth, the first row shows the results of standard GP, the next six rows correspond to PP with different PC values, and the final three rows show the results obtained using the adaptive PP method. Lower fitness values are preferred, as they indicate that fewer relocations were required when solving the test set. The best values obtained with PP are highlighted in bold. Statistical comparisons between GP without and with parsimony pressure for each PC value are reported alongside the median fitness and tree size.

The results for fitness demonstrate that GP with parsimony pressure (PP) generally achieves a slightly worse median fitness compared to standard GP. This is expected, as PP forces a trade-off between solution quality and tree size. However, statistical tests show no significant deterioration for the smallest PC value of 1. Even with this small PC, the resulting solutions are considerably smaller than those generated via standard GP, particularly for a tree depth of 7, where the median tree size is reduced by nearly 50%. As the PC value increases, fitness deteriorates more, being approximately 2.5% worse than standard GP for the largest values. Nevertheless, the size of the expressions can be drastically reduced to only 8 or 7 nodes, compared to 49 and 110 nodes for standard GP, representing a substantial improvement. By choosing intermediate PC values, a favourable compromise between solution quality and tree size can be achieved. For example, with PC = 10, fitness deteriorates by only 0.5%, while tree size decreases from 49 to 21 for maximum depth 5, and from 110 to 20 for maximum depth 7. For PC = 30, performance decreases by around 1%, yet tree size can be reduced to just 13 nodes in both cases. These results highlight that a small reduction in fitness can lead to a significant simplification of the expression trees.

For the adaptive PP variant, performance is strongly influenced by the smoothing factor. Smaller smoothing values result in better fitness, while larger values produce solutions with lower fitness but fewer nodes. For the smallest smoothing value, performance is slightly worse than that of standard GP or PP with PC = 1, but it achieves a smaller median tree size than both methods. This indicates that the adaptive approach can offer a favourable compromise between solution quality and tree size. However, for larger smoothing values, the resulting solutions are inferior to those obtained with fixed PC values in terms of both fitness and size. Overall, adaptive PP appears most suitable when the goal is to reduce tree size moderately without substantially affecting fitness.

Table 4 presents the results for the unrestricted CRP variant. Overall, the patterns are similar to those observed for the restricted variant, with one notable exception. For PC = 10, GP with parsimony pressure does not show a significant deterioration in fitness for either maximum tree depth. At the same time, it reduces the solution size to just 17 nodes, allowing a threefold reduction for maximum depth 5 and a sixfold reduction for maximum depth 7, without any meaningful loss in performance. This further illustrates that standard GP-generated expressions often contain many redundant elements that can be removed with a minimal impact on solution quality. For this problem variant, adaptive PP achieves significantly better fitness and smaller trees compared to standard GP when the smallest smoothing parameter is used. This demonstrates the effectiveness of the adaptive method in balancing solution quality and size, by dynamically adjusting the PC to reduce tree size without substantially affecting performance. However, as the smoothing factor increases, the method struggles to maintain this balance, highlighting the importance of selecting an appropriate value for the smoothing parameter.

**Figure 3:**
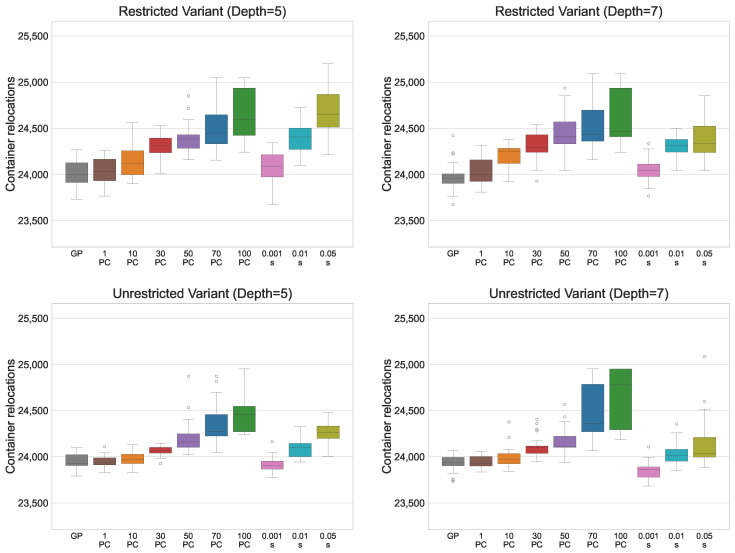
Box plots outlining the solution fitness (number of container relocations) under PP with fixed PC values, and for the adaptive PP method with smoothing factor *s* applied. The dots in the plot represent outliers.

Figure 3 presents the solution fitness in a box plot, illustrating the influence of different PC values on solution quality. For the restricted variant, performance deterioration occurs at smaller PC values compared to the unrestricted variant. Moreover, the largest deterioration in fitness occurs at the highest PC values, whereas in the restricted variant, the decline in performance is more gradual as PC increases. This suggests that the unrestricted variant is more resilient to reductions in solution size. A potential explanation for this behaviour is that the unrestricted RS performs some relocations independently of the PF, embedding part of the decision logic directly into the RS. As a result, the PF is relieved from encoding these decisions, allowing effective strategies to be represented with fewer nodes. In contrast, the restricted variant relies more heavily on the PF to compensate for the limited capabilities of the RS. Consequently, reductions in PF size directly constrain its ability to represent effective strategies, leading to earlier and more pronounced performance degradation.

For the adaptive variant, increasing the smoothing parameter generally leads to worse solutions. For the smallest smoothing value, adaptive PP achieves performance comparable to standard GP for the restricted variant and even surpasses GP for the unrestricted variant. This indicates that focusing the search on the space of smaller solutions is particularly beneficial for the unrestricted variant, suggesting that this space contains a higher density of high-quality solutions. Directing the search towards these smaller solutions enables GP to explore them more effectively and identify better strategies. Furthermore, since adaptive PP outperforms PP with a fixed PC value, this demonstrates the advantage of allowing the algorithm to explore both larger and smaller solutions. A likely explanation is that larger solutions provide more opportunities for GP to evolve high-quality subtrees or building blocks. These subtrees can later be reused and combined into smaller, more effective expressions. Adaptive PP allows the algorithm to explore both larger and smaller solutions, retaining diversity in the search space while ultimately producing compact solutions that maintain or even improve performance. This effect is especially pronounced in the unrestricted variant, where the relocation scheme already handles part of the decision logic, allowing smaller PFs to remain effective.

**Figure 4:**
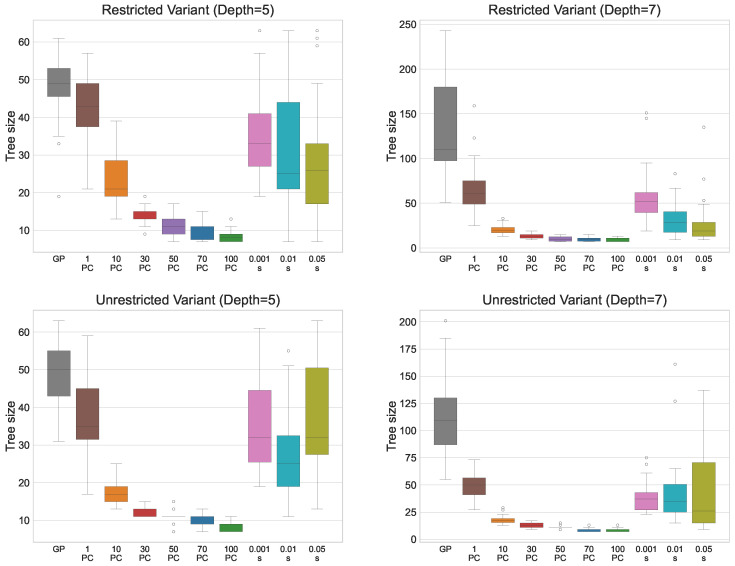
Box plots outlining the solution size (number of nodes) under PP with fixed PC values and for the adaptive PP method with smoothing factor *s* applied. The dots in the plot represent outliers.

Figure 4 presents the solution sizes obtained for different PC values. The reduction in size is similar for both the restricted and unrestricted variants, and for a given PC value, the median solution size is comparable across the two problem variants. Notably, for the larger maximum tree depth of 7, GP produces very large individuals without improving solution quality, clearly illustrating the effects of bloat. For adaptive PP, the median number of nodes is smaller than that of standard GP or PP with PC = 1 but remains larger than that obtained with higher fixed PC values, reflecting the balance it strikes between maintaining solution quality and reducing the tree size.

Regarding the reliability of the approach, increasing the PC value does not noticeably affect the dispersion of fitness values until the largest PC values of 70 and 100, for which variability significantly increases. This indicates that moderate increases in PC do not compromise the method’s ability to consistently obtain good solutions. For solution sizes, standard GP and PP with small PC values produce solutions with high variability. In contrast, larger PC values yield compact solutions with little variation, typically differing by only a few nodes. This demonstrates that increasing the PC allows the method to reliably produce consistently smaller solutions across runs. For the adaptive approach, the dispersion in fitness and solution size remains similar across all smoothing parameter values. While fitness variability is small, solution sizes vary considerably, indicating that adaptive PP is less reliable when the goal is to consistently obtain compact solutions.

Based on these observations, PP with a fixed PC value is most suitable when the primary goal is to reliably obtain consistently smaller solutions. Conversely, if the aim is to achieve high-quality solutions that are also more compact than those produced via standard GP, the adaptive method is preferable. By dynamically adjusting the PC during evolution, adaptive PP allows GP to maintain strong solution quality while still favouring smaller, more efficient solutions.

**Figure 5:**
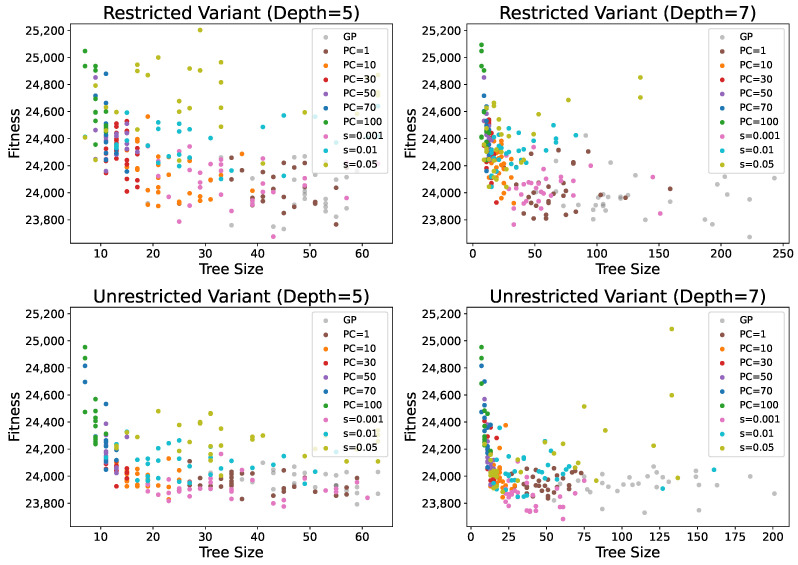
Distribution of solutions based on solution quality (number of container relocations) and size under parsimony pressure, compared to GP.

Figure 5 illustrates the distribution of results obtained across all tested methods, considering both solution quality and size. The figure confirms the previous observations, showing that smaller PC values (1, 10, and 30) are particularly beneficial since the increase in fitness is negligible compared to the reduction in solution size, especially for PC = 1. Higher PC values not only lead to larger fitness scores but also exhibit greater variability in fitness, making the results less predictable. The figure further highlights how the solution distribution changes with the PC parameter. For small PC values, solutions show low dispersion in fitness but high dispersion in solution size. As PC increases, the dispersion in fitness grows while the solution sizes become more consistent. These observations indicate that, with low or no parsimony pressure, GP tends to produce solutions of similar quality, but their sizes can vary considerably. Conversely, with high parsimony pressure, GP generates solutions with more consistent sizes, though their fitness may be more dispersed, reducing reliability in obtaining good solutions. Overall, intermediate PC values, such as 10 or 30, appear to provide the best trade-off between solution quality and size. At these values, GP achieves reliable results in terms of both compactness and performance, making them particularly effective choices for controlling bloat while maintaining solution quality.

For adaptive PP, the smoothing parameter again has a significant impact on the results. Larger smoothing values generally lead to worse performance compared to PP with a fixed PC value. In contrast, for the smallest smoothing parameter, the obtained results are comparable to those of other methods, and in the case of the unrestricted variant, they are often even better. However, the solution sizes for this parameter setting show high variability, indicating that adaptive PP provides less strict control over tree size compared to fixed PC parsimony pressure.

**Figure 6:**
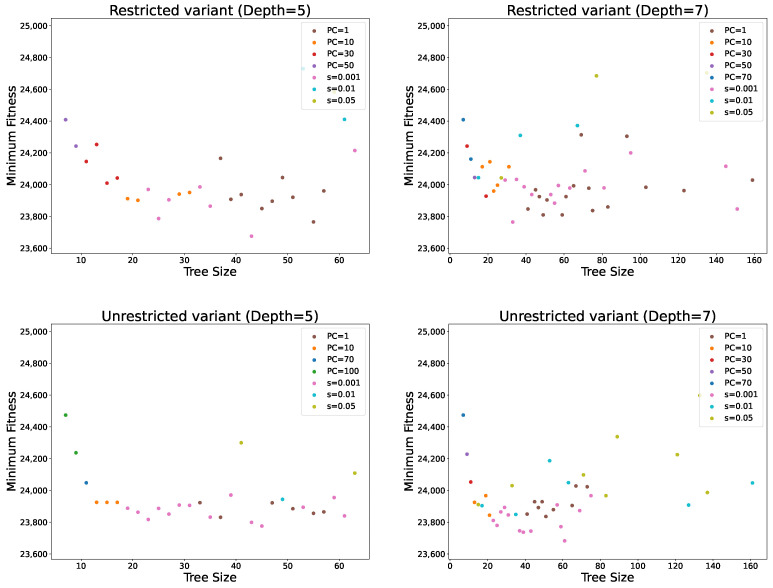
Best obtained solution fitness (the smallest number of container relocation) for each solution size under parsimony pressure.

Figure 6 shows the minimum fitness achieved with any of the PP methods for each obtained tree size. The figure confirms that PC = 1 achieves fitness values very similar to standard GP while producing good results across most solution sizes. It also demonstrates that using the three smallest PC values alone provides good coverage of both fitness and tree sizes, suggesting that there is generally no need to increase PC beyond 30 since similarly compact and high-quality solutions can be obtained with smaller PC values. An interesting observation is that, for very large solution sizes (over 100 nodes), solution quality deteriorates significantly. This reinforces the conclusion that excessively large solutions are unlikely to perform well, as they tend to contain many redundant or noisy elements. Furthermore, the adaptive PP variant achieves the best fitness values across many solution sizes, particularly for the smallest smoothing parameter. In most cases, it provides the best fitness for solution sizes between 20 and 40 nodes, highlighting its ability to balance solution quality and size effectively.

#### 5.2.2. Results for Expression Simplification

Table 5 presents the results obtained using the pruning method for different threshold values. Even at the smallest threshold, pruning causes a noticeable deterioration in fitness compared to standard GP. As expected, larger thresholds allow greater reductions in solution quality, with fitness deteriorating by up to approximately 13%. In all cases, pruning was able to significantly reduce the solution size, with slightly better performance observed for the maximum tree depth of 5. This suggests that removing redundancies in larger solutions may be more challenging than in smaller ones. Overall, the method’s performance is consistent across both considered maximum tree depths.

Table 6 presents the results for the unrestricted variant. The overall behaviour of the pruning method is similar to that observed for the restricted variant. Even at the smallest threshold value, a noticeable deterioration in solution quality occurs. However, compared to the restricted variant, pruning typically produces smaller solutions for the unrestricted case. This further supports the observation that, in the unrestricted variant, it is easier to obtain compact solutions that maintain good performance.

**Figure 7:**
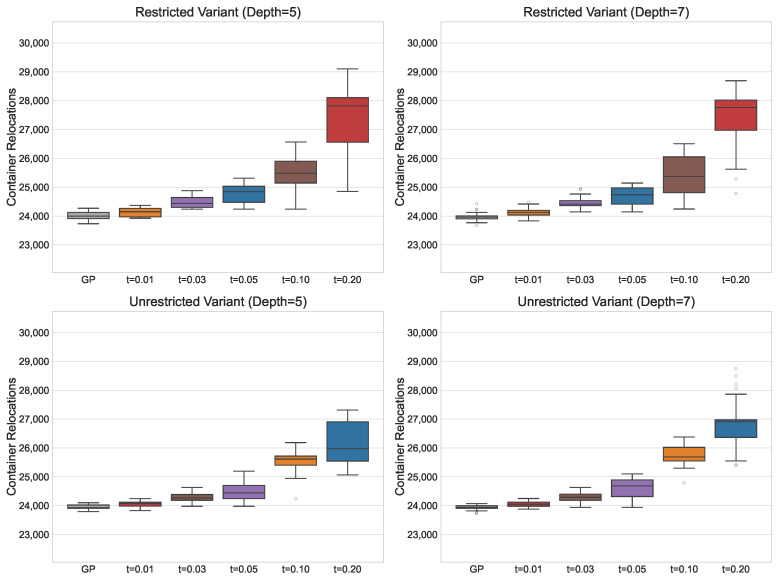
Box plot outlining the solution fitness (number of container relocations) under pruning for different threshold values.

**Figure 8:**
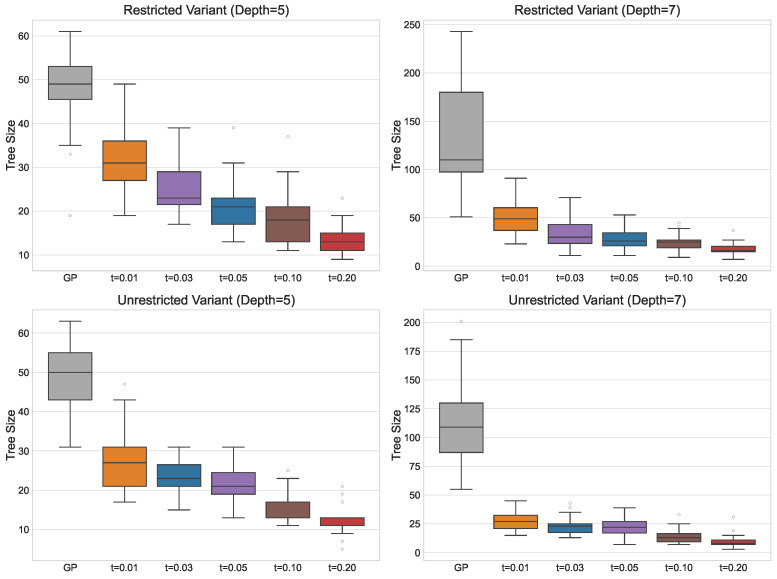
Box plot outlining the solution size (number of nodes) under pruning for different threshold values.

Figure 7 presents box plots of solution fitness for different pruning threshold values. As the threshold increases, solution quality steadily deteriorates, and the dispersion of fitness values grows, indicating that the method can reliably generate high-quality solutions. As noted previously, this deterioration is less pronounced for the unrestricted variant than for the restricted one, although the overall trends are similar across both variants. Still, for small thresholds, pruning produces solutions with low variability in fitness. Figure 8 shows the corresponding box plots for solution sizes. For the same threshold, the unrestricted variant consistently exhibits better distributions of solution sizes. For solution size, the trend is reversed: small thresholds lead to high variability in size, whereas larger thresholds reduce this variability. However, even for larger thresholds, some solutions can still be relatively large, showing that increasing the threshold does not guarantee a more reliable production of smaller solutions.

**Figure 9:**
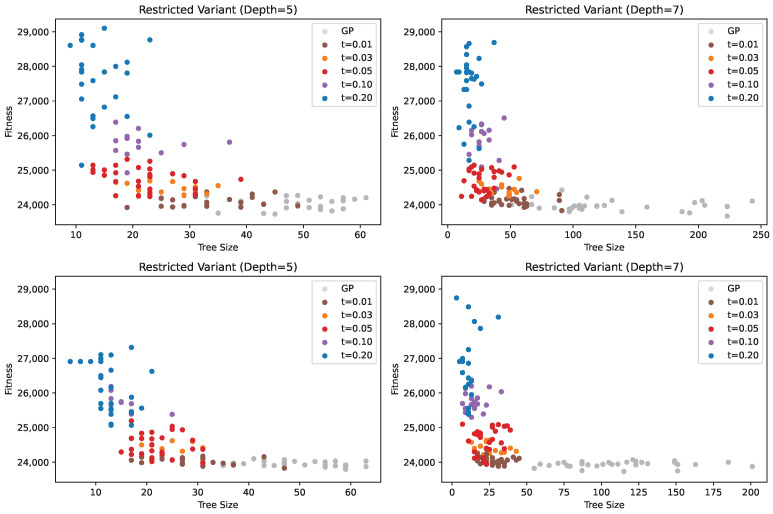
Distribution of solutions based on solution quality (number of container relocations) and size (number of nodes) under pruning.

Figure 9 illustrates the distribution of solution quality relative to the solution size. Solutions generated via standard GP achieve consistently good fitness values but are spread across a wide range of sizes, which are generally large. Applying pruning produces smaller solutions, though at the cost of reduced fitness. Interestingly, in some cases, increasing the pruning threshold does not further reduce solution size but only degrades performance. This effect is particularly pronounced for the maximum tree depth of 7. For instance, increasing the threshold from 0.05 to 0.10 does not yield smaller solutions. This behaviour reflects the mechanism of pruning; higher thresholds allow the acceptance of worse solutions, which are not necessarily more compact.

**Figure 10:**
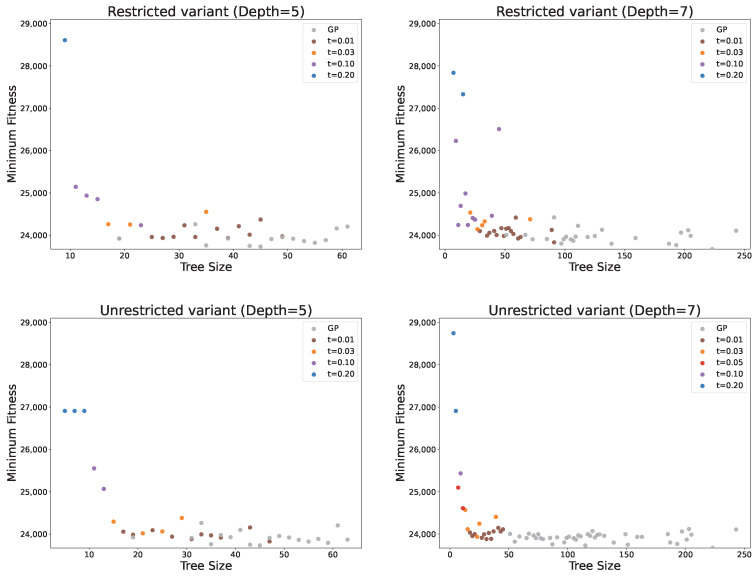
Best obtained solution fitness (number of container relocations) for each solution size (number of nodes) under pruning.

Figure 10 shows the best solutions obtained for each solution size. Most solutions correspond to the smallest threshold value, which is expected, as it imposes the least restriction on solution size. Interestingly, even with a moderately larger threshold of 0.03, some solutions of substantial size are still obtained, reflecting the same behaviour discussed previously. For the largest threshold values, the method can produce very compact solutions (below size 10), but this comes at the cost of a substantial deterioration in solution quality.

#### 5.2.3. Comparison Between Pruning and Parsimony Pressure

**Figure 11:**
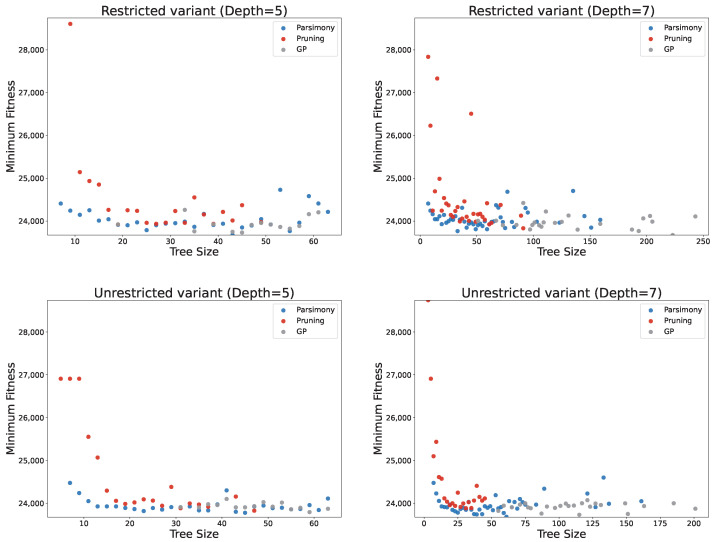
Distribution of solution quality and size for the best solutions obtained via pruning and parsimony pressure.

Figure 11 illustrates the distribution of solutions obtained via standard GP, GP with PP, and pruning. It is immediately evident that pruning generally yields the poorest results, as, for a given solution size, it produces solutions of lower quality. In contrast, GP with PP provides competitive results compared to standard GP. The only scenario in which GP with PP underperforms is for very large solutions, with 80 nodes or more. However, these large expressions offer minimal improvement in solution quality, indicating little incentive to use them. For smaller solution sizes, GP with PP often matches or even surpasses the performance of standard GP, demonstrating its effectiveness in generating compact yet high-quality solutions.

These results indicate that, within the experimental setting considered, parsimony pressure provides a more favourable trade-off between solution quality and size by integrating complexity control directly into the optimisation process. In contrast, pruning functions as a post hoc simplification technique and, therefore, cannot influence the structure of solutions during their construction. While this limits its effectiveness in the present context, pruning remains well suited for alternative applications, such as the retrospective simplification or analysis of already-evolved complex rules where retraining is impractical or impossible. Based on the results above, we conclude that GP with parsimony pressure is the more suitable approach to reducing solution complexity when compactness is a design objective during learning, as it maintains performance comparable to standard GP while explicitly enabling a controlled trade-off between solution quality and size.

## 6. Further Analysis

This section provides a more detailed analysis of the impact of the considered simplification methods on the complexity of the generated solutions, using various metrics derived from the expression tree size.

### 6.1. Analysis of Solution Complexity

This section presents additional measures used to more precisely quantify the complexity of the solutions generated via each approach. The measures considered are as follows:

- Average node count (NC)—the mean number of nodes per tree.
- Average tree depth (ND)—the mean depth of the trees.
- Average number of unique functions (UF)—the mean number of distinct function nodes appearing in each tree.
- Average number of unique terminals (UT)—the mean number of distinct terminal nodes appearing in each tree.
- Average number of subexpression duplicates (SDs)—the number of subexpressions appearing more than once within a tree.
- Average number of subexpression duplicates of size 3 (SD3)—the number of size 3 subexpressions that appear more than once in a tree, each consisting of two terminals and one operator. This measure specifically captures the repetition of the smallest syntactically valid operator-based subexpressions.
- Average number of repeated subexpressions (RS)—the total number of repeated subexpressions in a tree, counting all occurrences of each duplicated subexpression.
- Average number of repeated subexpressions of size 3 (RS3)—the total number of repeated subexpressions of size 3, each consisting of two terminals and one function node.

Table 7 summarises these statistics for a maximum tree depth of 5. For standard GP, the trees tend to be quite large in terms of node count. With a tree depth of 5, the maximum possible number of nodes is 63, and the average node count of the evolved expressions is about 80% of this maximum, indicating that the trees are mostly full. Simplification methods, however, can reduce this considerably: the most conservative methods decrease the node count by around 8, while the more aggressive methods can reduce it by up to 40 nodes on average. Regarding tree depth, standard GP typically produces trees that reach the maximum allowed depth. Parsimony pressure is more effective at reducing depth, with larger PC values lowering the average depth to 4 or even 3. In contrast, pruning has little effect on tree depth, and trees generally retain their maximum depth. This difference arises because it is more difficult to restructure an expression tree after evolution, whereas parsimony pressure continuously guides the GP search toward smaller, simpler solutions during the evolutionary process.

For unique functions and terminals, standard GP produces trees that use nearly all available primitives on average, with four function nodes and six terminal nodes. When parsimony pressure is applied, the average number of functions decreases to around three, dropping further to 2.6 for the largest PC values. The number of terminals is also reduced, by one for smaller PC values and by two to three for larger ones. Pruning is less effective at reducing the number of unique functions, with the average remaining above three even for the largest threshold values. However, it can reduce the number of terminals, by one for the smallest threshold and up to two to three for larger thresholds. Overall, the results suggest that both methods can act as feature selectors, removing unnecessary primitive nodes from the expressions and thereby simplifying the trees without substantially compromising solution quality.

Both methods are also effective at reducing duplicate subexpressions in the generated trees. Standard GP typically contains around four repeated subexpressions, usually with two distinct expressions being repeated, most of which are of size 3. Pruning, even with the smallest threshold values, reduces the number of duplicate subexpressions by an order of magnitude. For parsimony pressure, the two smaller PC values still leave at least one repeated subexpression on average, but for larger PC values, the number of repeated subexpressions drops to zero. The adaptive variant of PP similarly reduces duplicate subexpressions effectively. Overall, these results demonstrate that both pruning and parsimony pressure efficiently eliminate repeated subexpressions, contributing to a more compact and less redundant tree structure.

Table 8 summarises these statistics for a maximum tree depth of 7. As before, standard GP produces the largest trees on average. For this depth, the maximum possible number of nodes is 255, and the trees generated via GP typically reach about half of this maximum, indicating substantial growth. Both pruning and parsimony pressure are effective at reducing the node count and the tree depth, but PP proves slightly more efficient. For several PC values, PP produces trees with an average of around 10 nodes. Additionally, PP can reduce the tree depth to 3 or 4, whereas pruning generally only reduces it to around 6. This difference highlights a limitation of pruning: it is more constrained in reducing tree depth, likely because the evolved tree structures are already well adapted to their depth, making structural modifications without degrading solution quality more difficult.

For the number of unique functions and terminals, we observe a similar pattern as for depth 5. Pruning is slightly more effective at reducing the number of unique functions, whereas both methods achieve comparable reductions in the number of terminals. Typically, the number of unique functions is reduced by one or two nodes, while the number of unique terminals can be reduced to as few as three. This contributes to the overall simplicity of the solutions, as expressions that rely on fewer problem-specific terminals and operators are generally easier to interpret and understand.

Finally, we observe that standard GP generates trees with a large number of repeated subexpressions, highlighting potential redundancy in the evolved solutions. Both pruning and parsimony pressure are effective at reducing this redundancy: even for the smallest parameter values, the number of duplicate subexpressions is significantly lowered, while for larger parameter values, the generated trees contain virtually no repeated subexpressions.

From the above observations, it is clear that both simplification methods have a direct impact on multiple aspects of the generated solutions, altering their structure in different ways. Primarily, both methods reduce the total number of nodes, but this also influences other tree characteristics, including smaller depths, fewer redundant subexpressions, and a more selective use of the available building blocks, effectively highlighting the most important functions and terminals.

### 6.2. Analysis of Primitive Node Usage Frequency

**Figure 12:**
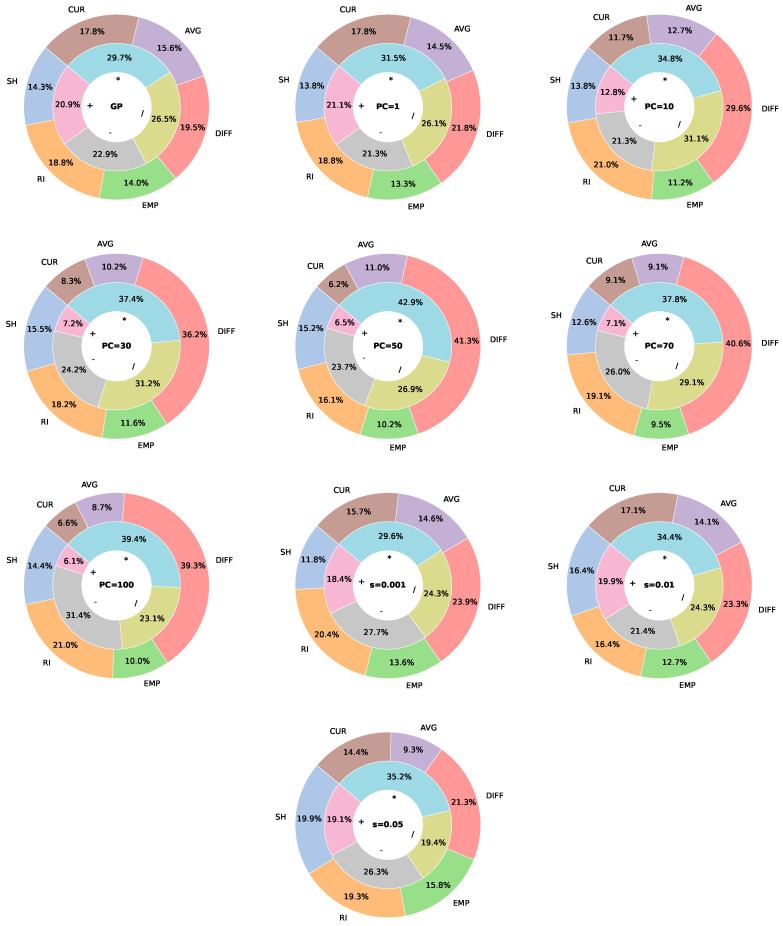
Usage frequency of terminals and functions for parsimony pressure compared to standard GP. Function node frequency is displayed in percentages on the inner ring, and terminal node frequency on the outer ring.

Figure 12 illustrates the usage frequency of terminals and functions when parsimony pressure is applied. For standard GP, all nodes are used relatively uniformly, with only minor differences in occurrence frequency. However, as PC increases, a clear shift in node usage emerges. Among the function nodes, the summation operator becomes less frequent, appearing in only about 7% of cases, while the multiplication operator dominates with roughly 40% usage. The division operator is also commonly used, at around 30%, whereas subtraction is slightly less frequent. Basically, this means that the PF tries to model the interaction between different terminals rather than simply summing their contributions. For terminal nodes, higher PC values lead to a concentration on specific nodes. In particular, DIFF becomes the most frequently used terminal, appearing in approximately 40% of instances, highlighting its importance in guiding relocation decisions. RI follows as the second most common terminal, with a frequency of around 20%. Together, these two terminals account for roughly 60% of all terminal occurrences, suggesting their importance in decision making. Conversely, CUR, AVG, and EMP are the least frequently used, appearing in no more than 10% of cases in most experiments. Thus, these terminals, although still important, have a smaller influence on the decision-making process. For the adaptive variant, we see that the distributions of the terminals and functions are the most similar to the distributions of PP with the smallest PC values.

**Figure 13:**
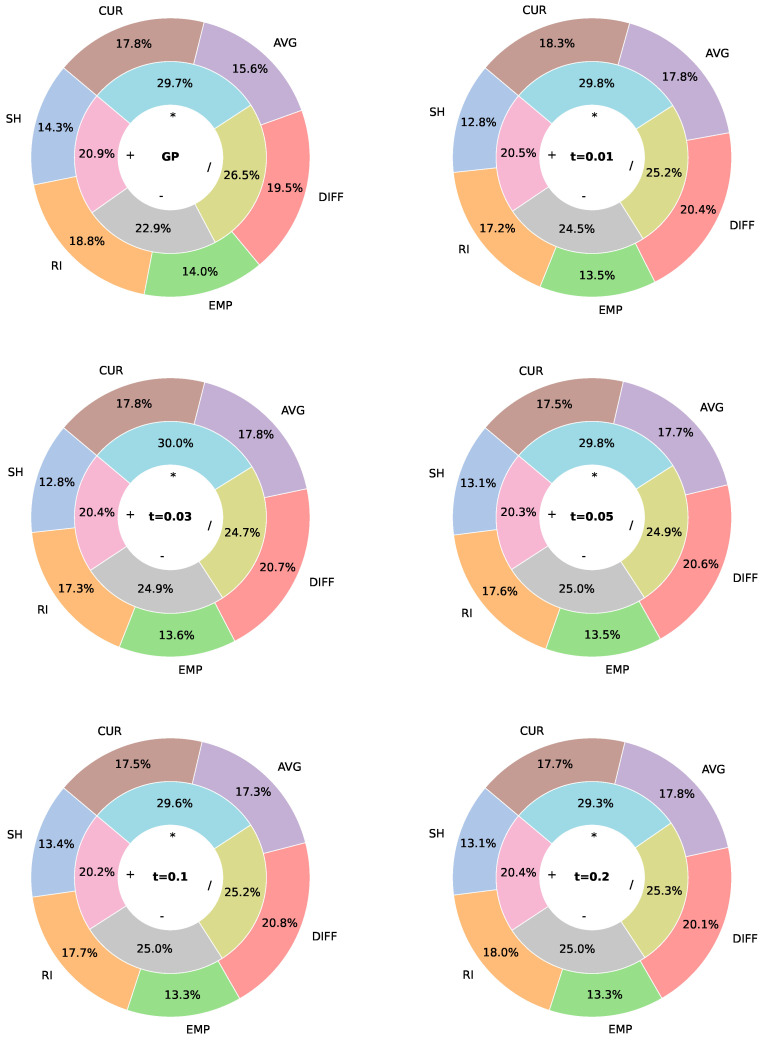
Usage frequency of terminals and functions for pruned trees compared to standard GP. The function node frequency is displayed in percentages on the inner ring, and terminal node frequency on the outer ring.

Figure 13 shows the occurrence frequency of primitive nodes when pruning is applied. Compared to standard GP, the usage of function nodes changes only slightly, and the shifts are less pronounced than those observed with parsimony pressure. As before, the frequency of the addition operator decreases, while multiplication and division become more dominant. Among terminal nodes, DIFF and RI continue to appear most frequently. In contrast, EMP consistently exhibits the lowest occurrence, whereas the remaining terminals show similar usage frequencies.

The previous results demonstrate that, by using the simplification methods, the composition of the trees changes significantly. More specifically, it seems that the methods try to reduce the reliance on additive operations, instead favouring multiplicative and divisive interactions between the most informative terminals, particularly DIFF and RI. This indicates that the PF prioritises modeling the relationships between key problem features, rather than combining all inputs indiscriminately, which likely contributes to more compact and focused expressions. At the same time, less informative terminals, such as EMP, are largely ignored, further streamlining the decision logic.

### 6.3. PF Examples

**Figure 14:**
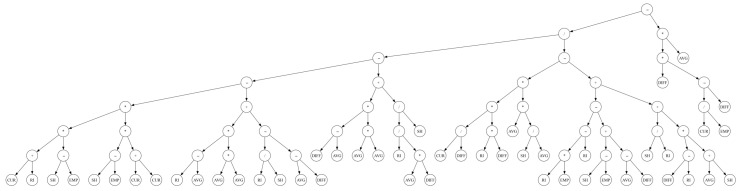
An example of an expression tree obtained via the standard GP algorithm.

**Figure 15:**
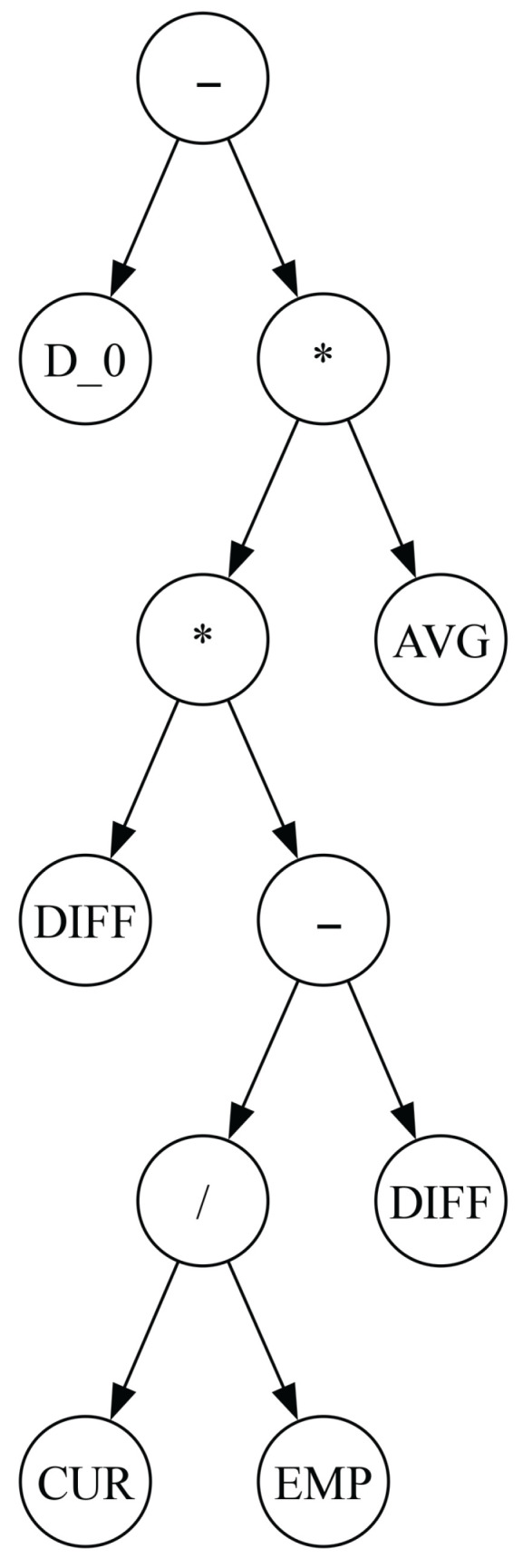
Expression tree shown in Figure 14 after applying pruning.

To gain insight into the amount of noise a solution can contain, it is instructive to examine a few obtained solutions. Figure 14 shows a solution generated via standard GP before any simplification. This solution has a fitness of 24,029 and contains 43 nodes. Applying pruning with a threshold of 0.1 yields the solution shown in Figure 15 (note that the node $D_{0}$ represents the constant 0). The pruned solution has a fitness of 24,936 and only 13 nodes, meaning that the fitness deteriorated by roughly 2%, while the tree size was reduced by a factor of three. Examining the result more closely, we observe that pruning removed the entire left subtree of the root, which contained 30 nodes. While the original expression was difficult to interpret, the pruned solution is now much more understandable. The RR makes decisions based on properties such as the number of empty spaces on a stack or the difference between the container being relocated and the container with the minimum ID on that stack. In essence, the rule prioritises stacks with more empty spaces where the relocated container will not block other containers.

**Figure 16:**
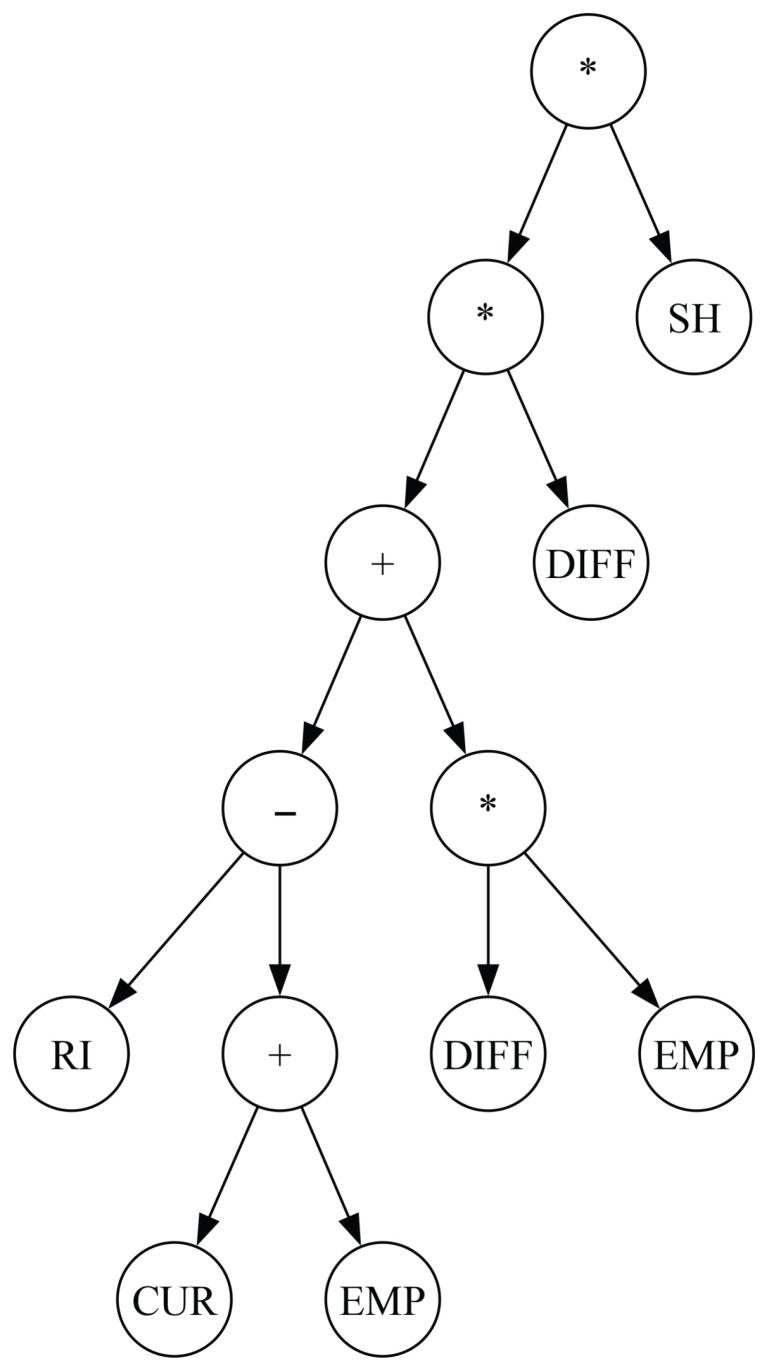
Expression tree obtained via GP with PP.

Figure 16 shows a solution obtained via GP with PP applied. This solution has a fitness value of 24,900 and contains 13 nodes, similar to the solution obtained via pruning. In this case, the expression is strongly influenced by stack height, as it multiplies the rest of the expression, which itself considers empty spaces, with an additive penalty. Consequently, the rule aims to select the stack with the fewest containers while also minimising the number of other containers that would be blocked by the relocated container.

## 7. Conclusions and Future Work

This study has investigated two methods of bloat control, parsimony pressure and pruning, applied to RRs automatically generated via GP for the container relocation problem. Typically, such simplification methods produce more compact solutions at the cost of some performance, as measured using the fitness of the resulting expressions. However, in many cases, the size of the expression tree can be as important as raw performance, since smaller and simpler expressions are easier to interpret, increasing user confidence and facilitating the potential extraction of new insights from the generated rules.

Both methods demonstrated their ability to reduce the solution size while having only a minimal effect on solution quality. However, parsimony pressure consistently produced solutions of the same size as pruning, but with better fitness. This can be attributed to the fact that parsimony pressure is an online method, integrated directly into the evolutionary process, which guides GP to focus its search on smaller solutions. In contrast, pruning is an offline method applied after GP has evolved a RR, meaning it cannot influence the search process and can only attempt to remove redundancies in the final solution. By that stage, the solution may already be overly complex, making it more difficult to eliminate redundant parts effectively. Both methods proved worthwhile for several parameter settings. In some cases, parsimony pressure even improved fitness while significantly reducing the tree size. Pruning never improved fitness, although the associated deterioration was relatively small, considering the reduction in size. Nevertheless, it cannot be concluded that parsimony pressure universally outperforms pruning, as their relative effectiveness depends on the chosen parameters, the nature of the problem, and the stochastic behaviour of the evolutionary process.

However, some limitations of this study should be noted. First, the evaluation was conducted solely on the Caserta dataset, so some observed behaviours may not generalise to other datasets or problem variants, particularly regarding the influence of parameter values on both performance and solution size. Second, only relatively simple metrics were used to assess the complexity and interpretability of the generated expressions. While these metrics provide an initial indication of structural simplification, they do not offer a comprehensive evaluation of interpretability. A more thorough assessment involving human experts would be necessary to determine whether the simplified expressions are genuinely understandable in practice. Finally, although the computational cost of the pruning procedure was discussed qualitatively, it was not quantitatively compared to the overall GP run time, leaving a detailed analysis as an important direction for future work.

The obtained results open several avenues for future research. Since the pruning method is computationally expensive, as each pruned solution must be evaluated, surrogate models could be employed to approximate the fitness of pruned expressions, thereby reducing computational costs. Lowering the complexity of pruning could also enable its integration directly into the GP process, allowing it to operate online, rather than offline, as in this study. This could potentially improve the effectiveness of pruning compared to GP with parsimony pressure. Although this study focused on obtaining simpler solutions, simplicity does not necessarily equate to interpretability. Therefore, a key research direction will involve defining and analysing solution-level measures related to interpretability, such as tree depth, weighted operator counts, input–output sensitivity, and the structural modularity of expression trees. Additionally, we plan to explore LLM-assisted evaluation frameworks, leveraging large language models to assess the semantic clarity, decomposability, and explanation quality of GP-generated solutions. 

## Figures and Tables

**Table 1:** Function and terminal sets used with GP for the CRP.

Symbol	Description
Terminals	
*SH*	Current height of the stack
*EMP*	Number of empty slots available in the stack
*CUR*	Identifier of the container currently being relocated
*RI*	Count of containers in the stack with an identifier smaller than that of the relocated container
*AVG*	Mean container identifier value within the stack
*DIFF*	Difference between the smallest container identifier in the stack and the identifier of the relocated container
Functions	
+	Summation operator
−	Subtraction operator
∗	Multiplication operator
/	Protected division (returns 1 if divisor is close to 0)

**Table 2:** GP parameter values used across all experiments. The maximum tree depth was fixed at the start of each run (either 5 or 7), and the same sets of crossover and mutation operators were used throughout, with operators selected at random at each iteration.

Parameter	Value
Population size	1000
Mutation probability	0.3
Maximum tree depth	5, 7
Selection operator	steady state tournament
Tournament size	3
Crossover operators	subtree, uniform, context-preserving, size-fair, and one-point
Mutation operators	subtree, hoist, node complement, node replacement, permutation, and shrink
Number of evaluations	50,000

**Table 3:** Statistics for solution quality (number of relocations) and tree size obtained for the restricted scheme using fixed parsimony coefficients (PC) and adaptive parsimony with smoothing factor applied (*s*). Symbols +, −, and ≈ indicate significant improvement, degradation, or no significant difference compared to GP without parsimony, respectively.

Depth	Method	Parameter		Number of Relocations			Tree Size		
				Min	Median	Max	Min	Median	Max
5	GP			23,732	23,999	24,269	19	49	61
	PP	PC	1	23,766	**24,032**≈	**24,259**	21	43+	57
			10	23,902	24,120−	24,563	13	21+	39
			30	24,010	24,312−	24,529	9	13+	19
			50	24,160	24,386−	24,852	**7**	11+	17
			70	24,157	24,450−	25,047	**7**	11+	15
			100	24,243	24,594−	25,047	**7**	**8**+	**13**
		*s*	0.001	**23,676**	24,091≈	24,344	19	33+	63
			0.01	24,102	24,407−	24,730	**7**	25+	63
			0.05	24,216	24,654−	25,202	**7**	26+	63
7	GP			23,673	23,961	24,423	51	110	243
	PP	PC	1	23,810	**23,997**≈	**24,316**	25	61+	159
			10	23,923	24,245−	24,383	13	20+	33
			30	23,928	24,288−	24,540	9	13+	19
			50	24,045	24,413−	24,936	**7**	9+	15
			70	24,161	24,439−	25,093	**7**	9+	15
			100	24,241	24,463−	25,093	**7**	**7**+	**13**
		*s*	0.001	**23,765**	24,049−	24,337	19	52+	151
			0.01	24,044	24,311−	24,499	9	28+	83
			0.05	24,043	24,340−	24,852	9	19+	135

**Table 4:** Statistics for solution quality (number of relocations) and tree size obtained for the unrestricted scheme using fixed parsimony coefficients (PC) and adaptive parsimony with smoothing factor applied (*s*). Symbols +, −, and ≈ indicate significant improvement, degradation, or no significant difference compared to GP without parsimony, respectively.

Depth	Method	Parameter		Number of Relocations			Tree Size		
				Min	Median	Max	Min	Median	Max
5	GP			23,792	23,932	24,099	31	50	63
	PP	PC	1	23,831	23,939≈	**24,110**	17	35+	59
			10	23,828	23,968≈	24,130	13	17+	25
			30	23,925	24,068−	24,145	11	13+	15
			50	24,023	24,159−	24,872	**7**	11+	15
			70	24,048	24,271−	24,872	**7**	**9**+	13
			100	24,237	24,452−	24,953	**7**	**9**+	**11**
		*s*	0.001	**23,776**	**23,903**+	24,164	19	32+	61
			0.01	23,944	24,102−	24,357	11	25+	55
			0.05	24,004	24,264−	24,480	13	32+	63
7	GP			23,730	23,940	24,071	55	109	201
	PP	PC	1	23,836	23,930≈	**24,058**	27	50+	73
			10	23,844	23,971≈	24,376	13	17+	29
			30	23,949	24,102−	24,408	9	13+	17
			50	23,937	24,109−	24,569	9	11+	15
			70	24,065	24,364−	24,953	**7**	9+	**13**
			100	24,186	24,778−	24,953	**7**	**7**+	**13**
		*s*	0.001	**23,683**	**23,862**+	24,109	23	37+	75
			0.01	23,848	24,017−	24,357	15	35+	161
			0.05	23,884	24,038−	25,087	9	26+	137

**Table 5:** Statistics for solution quality and size obtained for the restricted scheme when using tree pruning with different threshold values. The symbol + denotes that pruning with a certain threshold value achieved significantly better results compared to GP before pruning, while − indicates that it achieved worse ones.

		Number of Relocations			Tree Size		
Depth	Threshold	Min	Median	Max	Min	Median	Max
5	GP (none)	23,732	23,999	24,269	19	49	61
	0.01	**23,922**	**24,148**−	**24,371**	19	31+	49
	0.03	24,238	24,436−	24,877	17	23+	39
	0.05	24,238	24,846−	25,314	13	21+	39
	0.1	24,238	25,482−	26,568	11	18+	37
	0.2	24,853	27,822−	29,101	**9**	**13**+	**23**
7	GP (none)	23,673	23,961	24,423	51	110	243
	0.01	**23,832**	**24,129**−	**24,469**	23	49+	91
	0.03	24,144	24,410−	24,944	11	30+	71
	0.05	24,144	24,735−	25,144	11	26+	53
	0.1	24,243	25,371−	26,508	9	25+	45
	0.2	24,777	27,758−	28,689	**7**	**16**+	**37**

**Table 6:** Statistics for solution quality and size obtained for the unrestricted scheme when using tree pruning with different threshold values. The symbol + denotes that pruning with a certain threshold value achieved significantly better results compared to GP before pruning, while − indicates that it achieved worse ones.

		Number of Relocations			Tree Size		
Depth	Threshold	Min	Median	Max	Min	Median	Max
5	GP (none)	23,792	23,932	24,099	31	50	63
	0.01	**23,825**	**24,075**−	**24,245**	17	27+	47
	0.03	23,979	24,269−	24,634	15	23+	31
	0.05	23,979	24,442−	25,193	13	21+	31
	0.1	24,242	25,613−	26,185	11	13+	25
	0.2	25,067	25,976−	27,316	**5**	**13**+	**21**
7	GP (none)	23,730	23,940	24,071	55	109	201
	0.01	**23,882**	**24,048**−	**24,246**	15	27+	45
	0.03	23,937	24,291−	24,633	13	23+	43
	0.05	23,937	24,685−	25,098	7	22+	39
	0.1	24,793	25,687−	26,377	7	13+	33
	0.2	25,384	26,908−	28,744	**3**	**8**+	**31**

**Table 7:** The table shows averages of certain measurements for plain GP, parsimony pressure, and pruning, across all experiments that had the maximum tree depth set to 5. The measurements in question are in order: the node count (NC), the tree depth (ND), the number of unique function nodes (UF) and terminal nodes (UT), the number of all subexpression duplicates (SD) and those specifically of size 3 (SD3), and the number of all repeated subexpressions (RS) and those specifically of size 3 (RS3).

Method	Parameter	NC	ND	UF	UT	SD	SD3	RS	RS3
GP		48.7	5	3.9	5.8	2	1.8	4.3	3.9
pruning	0.01	29.5	5	3.9	4.8	0.2	0.2	0.5	0.5
	0.03	24.3	5	3.8	4.4	0.1	0.1	0.1	0.1
	0.05	21.3	5	3.7	4.1	0.1	0.1	0.2	0.2
	0.10	16.6	4.9	3.4	3.4	0	0	0	0
	0.20	13.2	4.8	3.1	2.8	0	0	0	0
parsimony	1	40.4	5	3.9	5.6	1.2	1.2	2.6	2.5
	10	20.5	4.7	3.6	4.8	0.5	0.4	1	0.9
	30	13.1	4.2	3.1	4.1	0	0	0	0
	50	10.9	3.8	2.9	3.6	0	0	0	0
	70	9.6	3.4	2.7	3.5	0	0	0	0
	100	8.3	3.1	2.6	3.3	0	0	0	0
	A-0.001	41	5	4	5	0.1	0.1	0.1	0.1
	A-0.01	29.4	4.8	3.6	5.2	0	0	0	0
	A-0.05	35.3	4.8	4	5.6	0.1	0.1	0.2	0.2

**Table 8:** The table shows averages of certain measurements for plain GP, parsimony pressure, and pruning, across all experiments that had the maximum tree depth set to 7. The measurements in question are in order: the node count (NC), the tree depth (ND), the number of unique function nodes (UF) and terminal nodes (UT), the number of all subexpression duplicates (SD) and those specifically of size 3 (SD3), and the number of all repeated subexpressions (RS) and those specifically of size 3 (RS3).

Method	Parameter	NC	ND	UF	UT	SD	SD3	RS	RS3
GP		121.6	7	4.0	6.0	7.6	7.1	17.0	15.9
pruning	0.01	38.6	6.6	3.8	5.4	0.4	0.4	0.9	0.9
	0.03	28.3	6.4	3.7	4.9	0.1	0.1	0.2	0.2
	0.05	25.5	6.3	3.7	4.7	0.1	0.1	0.3	0.3
	0.10	18.8	5.6	3.4	4.0	0	0	0	0
	0.20	13.5	5.0	2.8	3.0	0	0	0	0
parsimony	1	57.8	6.9	4.0	5.8	2.3	2.1	4.7	4.4
	10	19.6	5.7	3.5	4.5	0.3	0.2	0.6	0.5
	30	12.9	4.4	3.0	4.0	0.1	0.1	0.1	0.1
	50	10.6	3.6	2.9	3.8	0	0	0	0
	70	9.3	3.5	2.8	3.6	0	0	0	0
	100	8.3	3.2	2.6	3.5	0	0	0	0
	A-0.001	42.5	6.8	4.0	5.6	0.2	0.2	0.4	0.4
	A-0.01	43.2	6.5	3.7	5.2	0.1	0.1	0.2	0.2
	A-0.05	51.0	5.8	3.2	5.1	0.2	0.2	0.5	0.5

## Data Availability

Data will be made available on request.
